# Lovastatin upregulates microRNA-29b to reduce oxidative stress in rats with multiple cardiovascular risk factors

**DOI:** 10.18632/oncotarget.14462

**Published:** 2017-01-03

**Authors:** Fu Wang, Hui Ma, Wen-Jing Liang, Jing-Jing Yang, Xue-Qing Wang, Mei-Rong Shan, Yuan Chen, Min Jia, Ya-Ling Yin, Xue-Ying Sun, Jia-Ning Zhang, Qi-Sheng Peng, Yu-Guo Chen, Li-Ying Liu, Peng Li, Tao Guo, Shuang-Xi Wang

**Affiliations:** ^1^ The Key Laboratory of Cardiovascular Remodeling and Function Research, Qilu Hospital, Shandong University School of Medicine, Jinan, China; ^2^ Department of Rehabilitation Medicine, Jinan Municipal Hospital of Traditional Chinese Medicine, Jinan, China; ^3^ Department of Pharmacology, College of Pharmacy, Xinxiang Medical University, Xinxiang, China; ^4^ Department of Pharmacology, Pharmaceutical College, Central South University, Changsha, China; ^5^ Biology and Chemistry, Denison University, Granville, OH, USA; ^6^ Key Laboratory for Zoonosis Research, Ministry of Education, Institute of Zoonosis, Jilin University, Changchun, China

**Keywords:** lovastatin, microRNA-29b, oxidative stress, PA200, endothelial dysfunction, Pathology Section

## Abstract

**Aims:**

Proteasome-linked oxidative stress is believed to cause endothelial dysfunction, an early event in cardiovascular diseases (CVD). Statin, as HMG-CoA reductase inhibitor, prevents endothelial dysfunction in CVD. However, the molecular mechanism of statin-mediated normalization of endothelial function is not completely elucidated.

**Methods and Results:**

Lovastatin time/dose-dependently increased miR-29b expression and decreased proteasome activity in cultured human umbilical vein endothelial cells (HUVECs). Anti-miR-29b or overexpression of PA200 abolished lovastatin-induced inhibition of proteasome activity in HUVECs. In contrast, pre-miR-29b or PA200 siRNA mimics these effects of lovastatin on proteasome activity. Lovastatin inhibited oxidative stress induced by multiple oxidants including ox-LDL, H_2_O_2_, TNFa, homocysteine thiolactone (HTL), and high glucose (HG), which were reversed by inhibition of miR-29b in HUVECs. *Ex vivo* analysis indicated that lovastatin normalized the acetylcholine-induced endothelium-dependent relaxation and the redox status in isolated rat aortic arteries exposure to multiple cardiovascular risk factors. *In vivo* analysis revealed that administration of lovastatin remarkably suppressed oxidative stress and prevented endothelial dysfunction in rats with hyperglycemia, dyslipidemia, and hyperhomocysteinemia, as well as increased miR-29b expressions, reduced PA200 protein levels, and suppression of proteasome activity in aortic tissues.

**Conclusion:**

Upregulation of miR-29b expression is a common mechanism contributing to endothelial dysfunction induced by multiple cardiovascular risk factors through PA200-dependent proteasome-mediated oxidative stress, which is prevented by lovastatin.

## INTRODUCTION

Lovastatin is used for the treatment of hypercholesterolemia because it inhibits HMG-CoA reductase, and thus prevents HMG-CoA's conversion into mevalonic acid [1]. Independent of cholesterol-lowering activity, there are increasing evidences that statins have a potential benefit against cardiovascular diseases (CVD). Oxidative stress is believed to cause endothelial dysfunction, an early event and a hallmark in CVD, including atherosclerosis and hypertension [2–4]. At this standpoint, it is widely accepted that statins prevent endothelial dysfunction by decreasing oxidative stress in vascular cells. However, the targets for stain-reduced oxidative stress in CVD have not been completely elucidated.

Our and other's previous studies have proven that activation of proteasome links to oxidative stress and consequent endothelial dysfunction in CVD [4–6]. Proteasomes are responsible for the degradation of a large number of protein targets throughout eukaryotic cells. The function of proteasomes is regulated by activators that bind to the ends of the 20S core catalytic particle in a single or double fashion and open the ends of the core proteasome to allow entry of protein targets. As one of proteasome activators (PAs), PA200 is broadly expressed in mammalian tissues and it has been suggested to play a crucial role in response to DNA damage [7, 8].

MicroRNA (miRs) are approximately 20-nucleotide, single-stranded RNA molecules that target mRNA through partial complementarity and regulate gene expression through inhibition of translation or transcript degradation. Recently, Anna S *et al* reported that microRNAs plays an important role in the regulation of proteasome activity [9]. Furthermore, miR-29b replacement inhibits proteasomes in myeloma cells by targeting PSME4 that encodesPA200 [10]. Available data suggest that statin, such as lovastatin, exerts its effects by inhibiting the proteasome activity in osteoblast, and vascular endothelial and smooth muscle cells [11, 12]. Thus, it is interesting to establish if statin via regulation of miR-29b inhibits proteasome activity in CVD.

In the current work, we characterized how lovastatin regulates PA200 and determined the importance of miR-29b in oxidative stress-mediated CVD. Here we report that lovastatin increases miR-29b, resulting in reduction of PA200 and consequent oxidative stress and endothelial dysfunction in rat models of diabetes, dyslipidemia, and hyperhomocysteinemia.

## RESULTS

### Lovastatin increases miR-29b expression in endothelial cells

**Figure 1:**
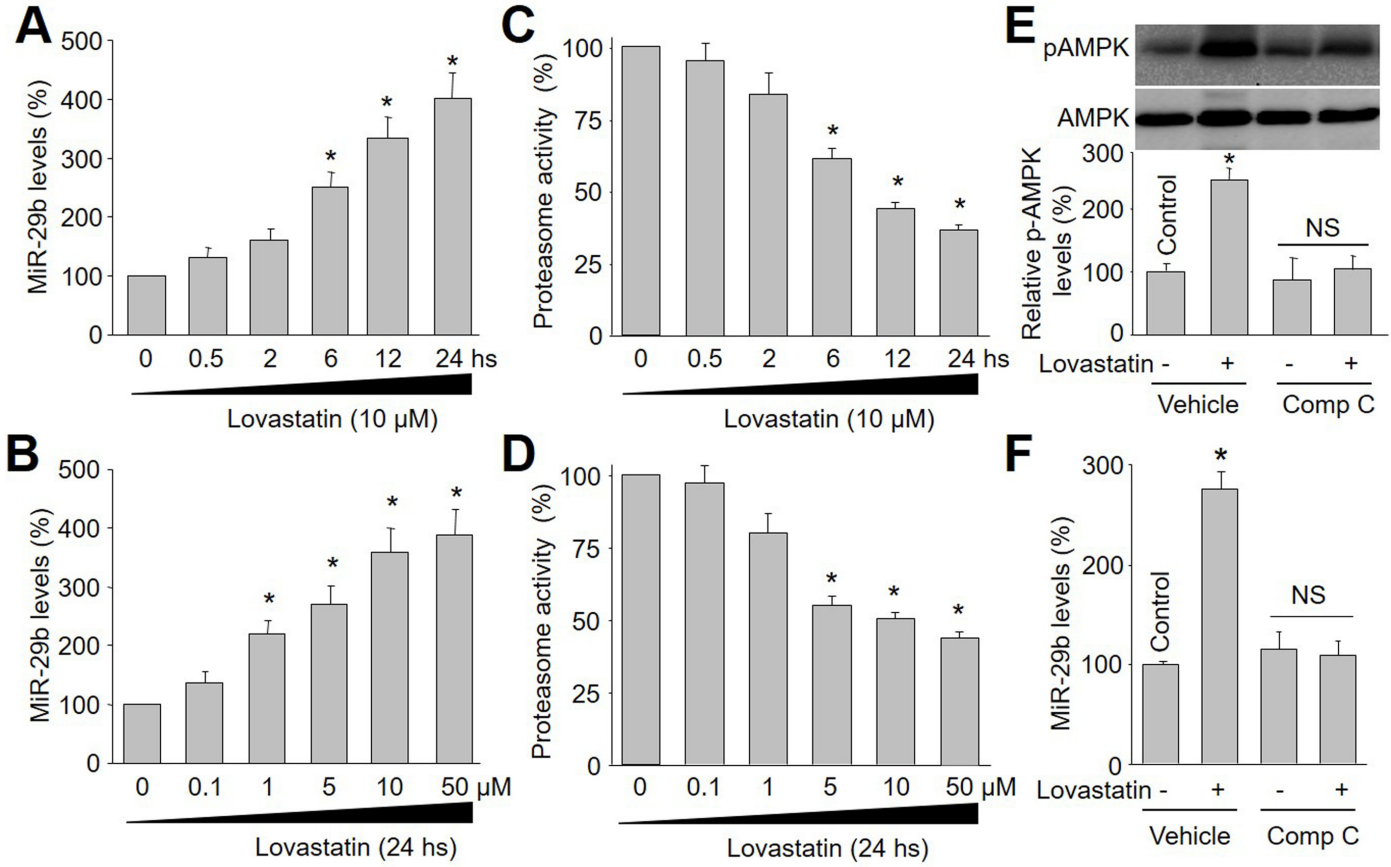
Lovastatin increases miR-29b expression and decreases proteasome activity in endothelial cells, which is in time/dose-dependent manner Cultured HUVECs were incubated with lovastatin as indicated time or concentration. HUVEC cell lysate was subjected to perform qRT-PCR analysis of miR-29b and measurement of proteasome activity. (**A**) Time course of lovastatin (10 μM) in miR-29b expression. (**B**) Dose course of lovastatin (24 hours) in miR-29b expression. (**C**) Time course of lovastatin in proteasome activity. (**D**) Dose course of lovastatin in proteasome activity. Data were expressed by mean ± sem. N is 3 in each group. *P < 0.05 *VS* Control group (Point 0). (**E** and **F**) HUVECs were incubated with compound C (Comp C, 1 μM) for 30 minutes followed by lovastatin (10 μM) treatment for 24 hours. The levels of p-AMPK in E and miR-29b in F were analyzed by western blot and RT-qPCR, respectively. N is 3 in each group. *P<0.05 VS Control. NS indicates no significance.

To investigate whether lovastatin increases miR-29b expression in endothelial cells, we firstly treated cultured HUVECs with varying time-points of lovastatin (10 μM) for 0.5 to 24 hours. As shown in Figure 1A, the levels of miR-29b gradually increased beginning from 6 hours and reached peak levels at 24 hours in endothelial cells.

The dose-dependent effects of lovastatin on miR-29b expression were next examined in endothelial cells. In Figure 1B, lovastatin at 1 μM significantly enhanced miR-29b expression. Increasing concentrations of lovastatin on (5-50 μM) further enhanced miR-29b expression. These observations indicate that lovastatin increases miR-29b expression in endothelial cells, which is in time- or dose-dependent manner.

### Lovastatin decreases proteasome activity in endothelial cells

The impacts of lovastatin on the proteasome activity *in vitro* were determined by exposing endothelial cells to lovastatin as indicated concentrations or times. As depicted in Figure 1C, lovastatin at 10 μM started to decrease proteasome activity at 6 hours to 24 hours. Similarly, 24-hour incubation of lovastatin at 5-50 μM markedly reduced proteasome activity in endothelial cells (Figure 1D), consistent with previous reports [11–15]. Based on these observations, 10 μM and 24-hour incubations of lovastatin were selected in most of the following experiments in endothelial cells.

### Lovastatin via AMPK activation increases the levels of miR-29b in endothelial cells

Lovastatin has been reported to activate AMPK via phosphorylation in endothelial cells [16]. Thus, we hypothesized that lovastatin via AMPK activation upregulates miR-29b expression in endothelial cells. To test this notion, we treated cells with compound C to inhibit AMPK activity. As shown in Figure 1E and 1F, lovastatin upregulated both miR-29b and p-AMPK expressional levels in cells treated with vehicle (DMSO), but not in cells treated with compound C, demonstrating that lovastatin via AMPK activation upregulated miR-29b expression in HUVECs.

### MiR-29b mediates lovastatin-induced suppression of proteasome activity in endothelial cells

**Figure 2:**
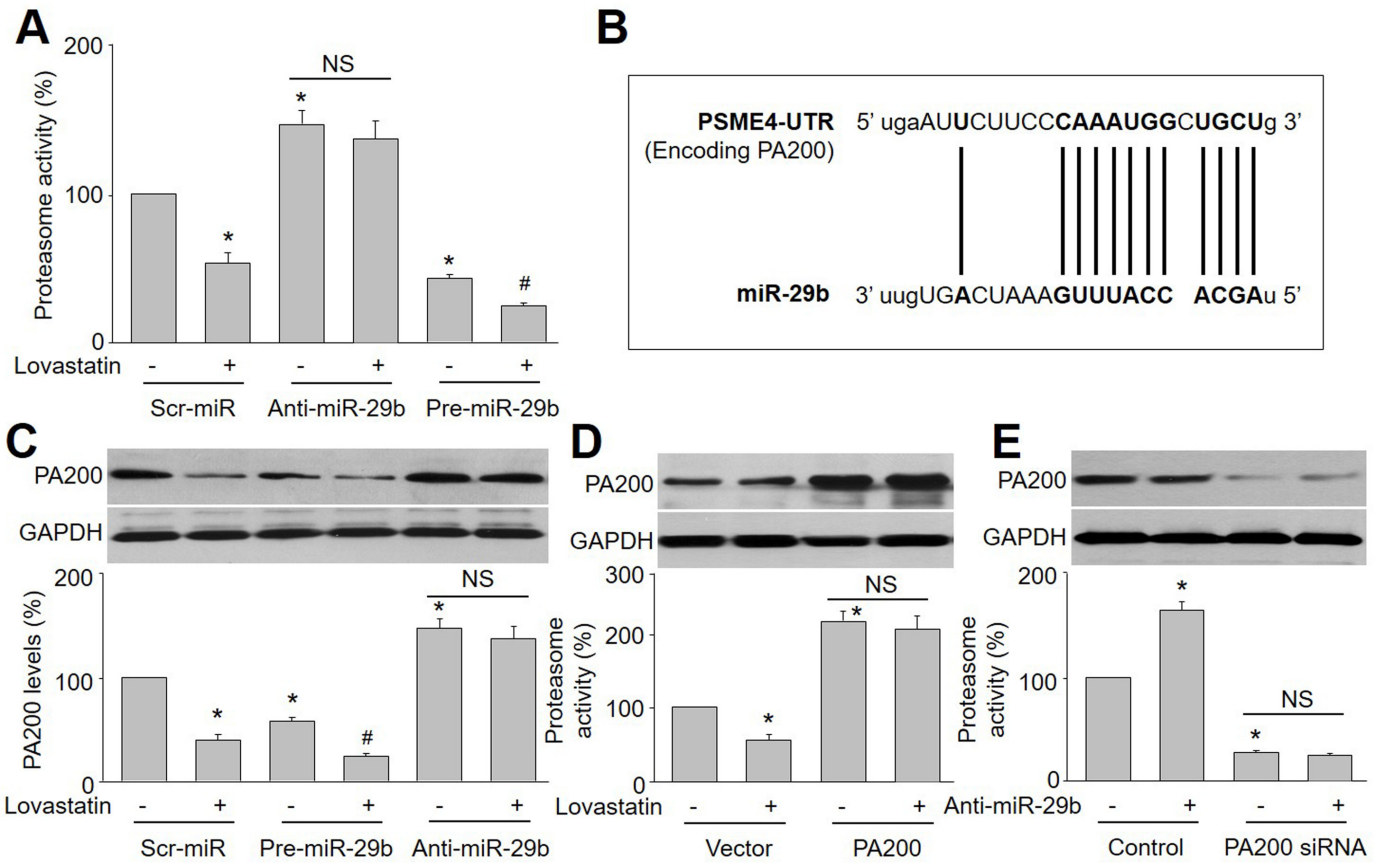
PA200 is a target of miR-29b to regulate proteasome activity in lovastatin-treated endothelial cells (**A**) Cultured HUVECs were infected with lentivirus expressing scrambled microRNA, anti-miR-29b and pre-miR-29b, and then incubated with lovastatin (10 μM) for 24 hours. Cell lysate was subjected to assay proteasome activity by measuring ATP-dependent degradation of proteasome fluorescence substrate. (**B**) The predictions of miR-29b targeting 3′-UTR of PSME4 by Target Scan. (**C**) PA200 protein expression was detected by Western blotting in cells from A. Data were expressed by mean ± sem. N is 3 in each group. *P < 0.05 *VS* Control or scr-miR group. ^#^*P* < 0.05 *VS* Pre-miR-29b alone group. (**D** and **E**) HUVECs were infected with (**D**) lentivirus containing PA200 cDNA or (**E**) transfected with PA200 siRNA, and then incubated with lovastatin (10 μM) for 24 hours. Cell lysate was subjected to assay proteasome activity. N is 3 in each group. *P < 0.05 *VS* Vector or scr-miR group. NS indicates no significant.

To investigate whether miR-29b mediates the suppression of lovastatin on proteasome activity in endothelial cells, HUVECs were infected with lentivirus expressing scrambled microRNA, anti-miR-29b and pre-miR-29b. As presented in Figure 2A, lovastatin still dramatically reduced proteasome activity in HUVECs infected with scr-miR lentivirus, but not in endothelial cells infected with anti-miR-29b lentivirus. The suppression of lovastatin on proteasome activity was further enhanced if cells were overexpressed with miR-29b. This indicates that the reduction of proteasome activity induced by lovastatin is mainly due to upregulation of miR-29b in endothelial cells.

### PA200 is a target of miR-29b in endothelial cells treated with lovastatin

PA200 has been identified as a target of miR-29b through PSME4 gene that encodes [10]. Thus, it was interesting to evaluate if lovastatin via upregulation of miR-29b reduces PA200 gene expression in endothelial cells. By performing Target Scan, we predicted the 3′-UTR of PSME4 as a miR-29b target (Figure 2B). Further, lovastatin significantly decreased PA200 protein level in endothelial cells. However, the downregulation of PA200 induced by lovastatin was abolished by anti-miR-29b and further amplified by overexpression of pre-miR-29b in HUVECs (Figure 2C), suggesting that a decreased PA200 by lovastatin may attribute to miR-29b.

### Inhibition of proteasome activity by lovastatin is PA200-dependent

PA200 is broadly expressed in mammalian cells, which is a proteasome activator [7, 10, 17]. We hypothesized that reduction of PA200 by lovastatin inhibits proteasome activity. To test this notion, endothelial cells were infected with lentivirus containing PA200 cDNA to establish PA200-overexpressed cell model (Figure 2D). Although lovastatin reduced proteasome activity in vector-infected cells, it failed to inhibit proteasome activity in endothelial cells with overexpression of PA200.

The role of PA200 in lovastatin-induced miR29b-mediated inhibitory effects on proteasome activity was further confirmed by decreasing the expression of PA200. As shown in Figure 2E, knockdown of PA200 mirrored the effects of lovastatin on suppression of proteasome activity. Anti-miR-29b, as an inhibitor of miR-29b, increased proteasome activity, which is opposite to lovastatin. However, anti-miR-29b did not alter proteasome activity in HUVECs if PA200 is deficiency by siRNA inference. Collectively, these data suggest that PA200 mediates the inhibition of proteasome activity by miR-29b in lovastatin-treated cells.

### Blockage of miR-29b abolishes the effects of lovastatin on suppressing ROS productions induced by multiple cardiovascular risk factors

**Figure 3:**
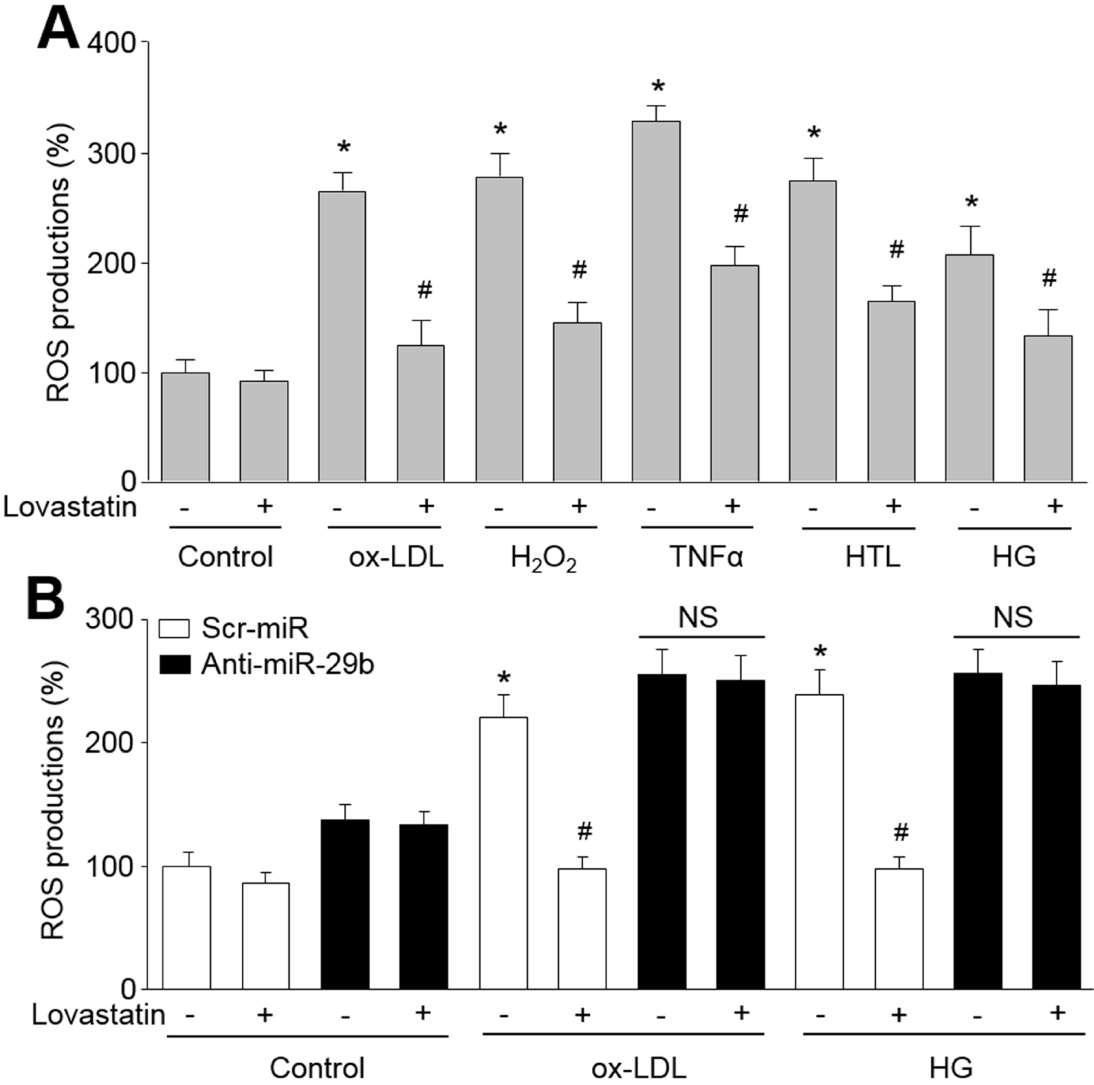
Lovastatin via upregulation of miR-29b suppresses oxidative stress in endothelial cells treated with oxidized low density lipoprotein (ox-LDL), hydrogen peroxide (H2O2), tumor necrosis factor alpha (TNFα), homocysteine thiolactone (HTL) or high glucose (HG) (**A**) Cultured HUVECs were pretreated lovastatin (10 μM) for 30 minutes followed by co-incubation with ox-LDL (100 mg/l), H_2_O_2_ (50 μM), TNFα (10 ng/ml), HTL (1 mM) or HG (30 mM) for 24 hours. ROS productions were determined by measuring DHE fluorescent intensity. Data were expressed by mean ± sem. N is 3 in each group. *P < 0.05 *VS* Control group. ^#^*P* < 0.05 *VS* ox-LDL, H_2_O_2_, TNFα, HTL or HG alone group. (**B**) HUVECs were infected with lentivirus containing with scrambled microRNA, anti-miR-29b and pre-miR-29b, and then incubated with lovastatin (10 μM) for 30 minutes followed by ox-LDL (100 mg/l) or HG (30 mM) for 24 hours. ROS productions were determined by measuring DHE fluorescent intensity. Data were expressed by mean ± sem. N is 3 in each group. *P < 0.05 *VS* scrambled microRNA alone group. ^#^*P* < 0.05 *VS* scrambled microRNA plus ox-LDL or HG alone group. NS indicates no significant.

We previously have reported that increased proteasome activity degrades IKBα or GTP cyclohydrolase I to induce oxidative stress in endothelial cells [4, 5]. We next determined whether suppression of proteasome activity inhibits oxidative stress in endothelial cells. As shown in Figure 3A, multiple cardiovascular risk factors, including ox-LDL, H_2_O_2_, TNFα, HTL and HG, dramatically increased ROS productions by measuring DHE fluorescent density in endothelial cells. As expected, Treatment with lovastatin reversed these abnormalities.

To investigate the role of miR-29b in lovastatin-suppressed oxidative stress, we tested the effects of lovastatin in cells deficient of miR-29b. As shown in Figure 3B, lovastatin reduced ROS productions induced by ox-LDL or HG in scr-miR-infected cells. Importantly, these suppressions were abolished in cells infected with anti-miR-29b lentivirus. In sum, these data suggest that upregulation of miR-29b by lovastatin suppresses oxidative stress in endothelial cells.

### Lovastatin prevents endothelial dysfunction induced by multiple oxidants in isolated rat descending aortic arteries *ex vivo*

**Figure 4:**
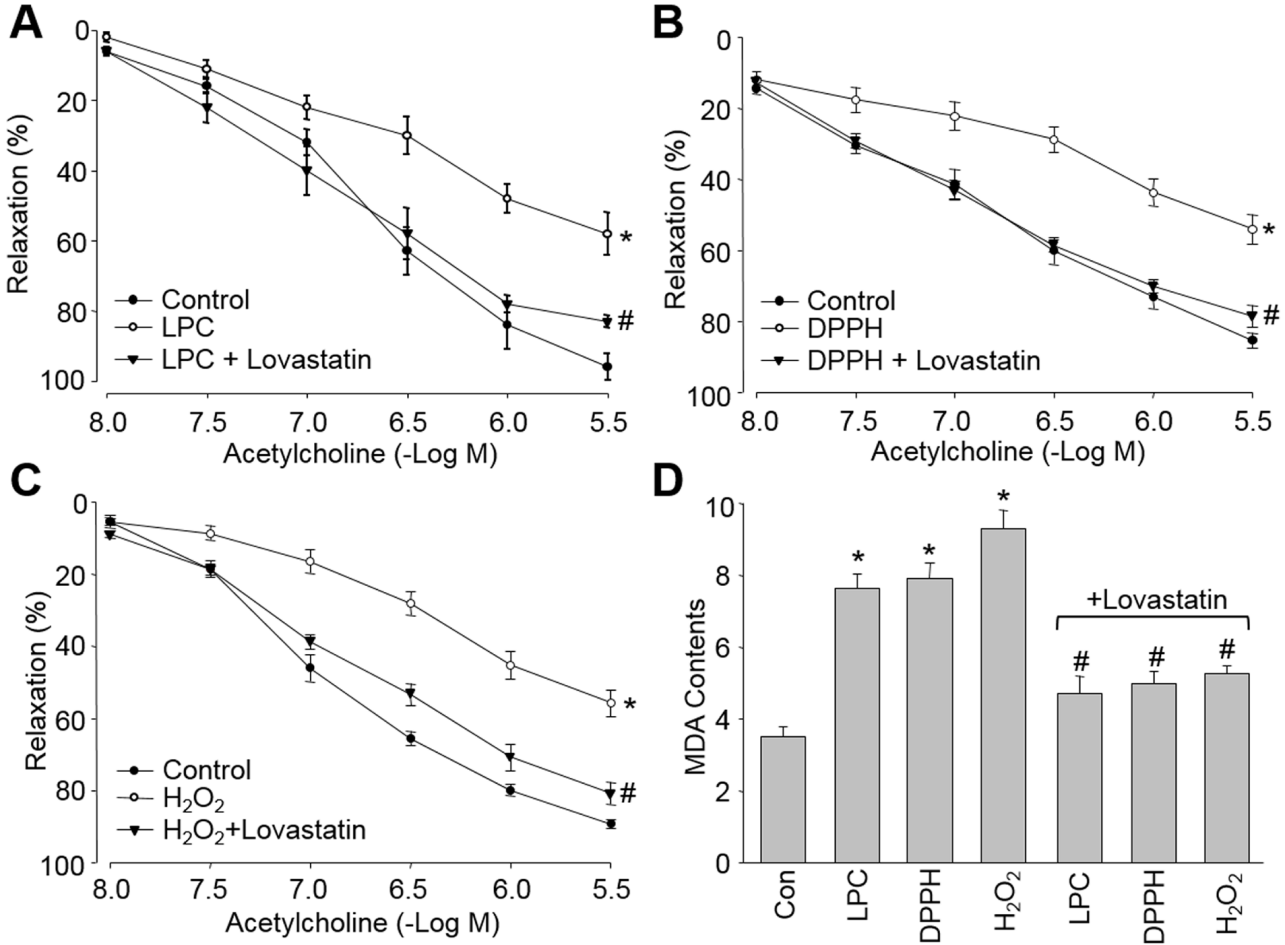
Lovastatin prevents endothelial dysfunction induced by multiple oxidants in isolated rat descending aortic arteries The isolated rat aortic rings were preincubated with lovastatin (10 μM) for 30 minutes and then exposed to (**A**) LPC (4 mg/l), (**B**) DPPH (1 μM), and (**C**) H_2_O_2_ (50 μM) for 2 hour. The endothelium-dependent relaxation induced by acetylcholine was assayed by organ chamber. (**D**) MDA content by TBA method. All data were expressed as mean ± SEM. N is 5 in each group. *P < 0.05 *VS* Control group. ^#^*P* < 0.05 *VS* LPC, DPPH or H_2_O_2_ alone.

Oxidative stress is believed to cause endothelial dysfunction, which is defined by impaired endothelium-dependent relaxation, is an early marker for CVD [2]. Thus, it was interesting to evaluate if lovastatin prevents endothelial dysfunction. We first tested this on *ex vivo* experiments in isolated rat descending aortic arteries. By incubations of rat aortic rings with LPC of the major metabolite of ox-LDL (Figure 4A), DPPH of an exogenous free radical (Figure 4B), and H_2_O_2_ of an endogenous ROS (Figure 4C), all oxidants impaired acetylcholine-induced endothelium-dependent relaxation. Pre-incubation of aortic rings with lovastatin bypassed these detrimental effects induced by oxidants including LPC, DPPH and H_2_O_2_ (Figure 4D).

### Lovastatin improves endothelial dysfunction in dyslipidemia rats

**Figure 5:**
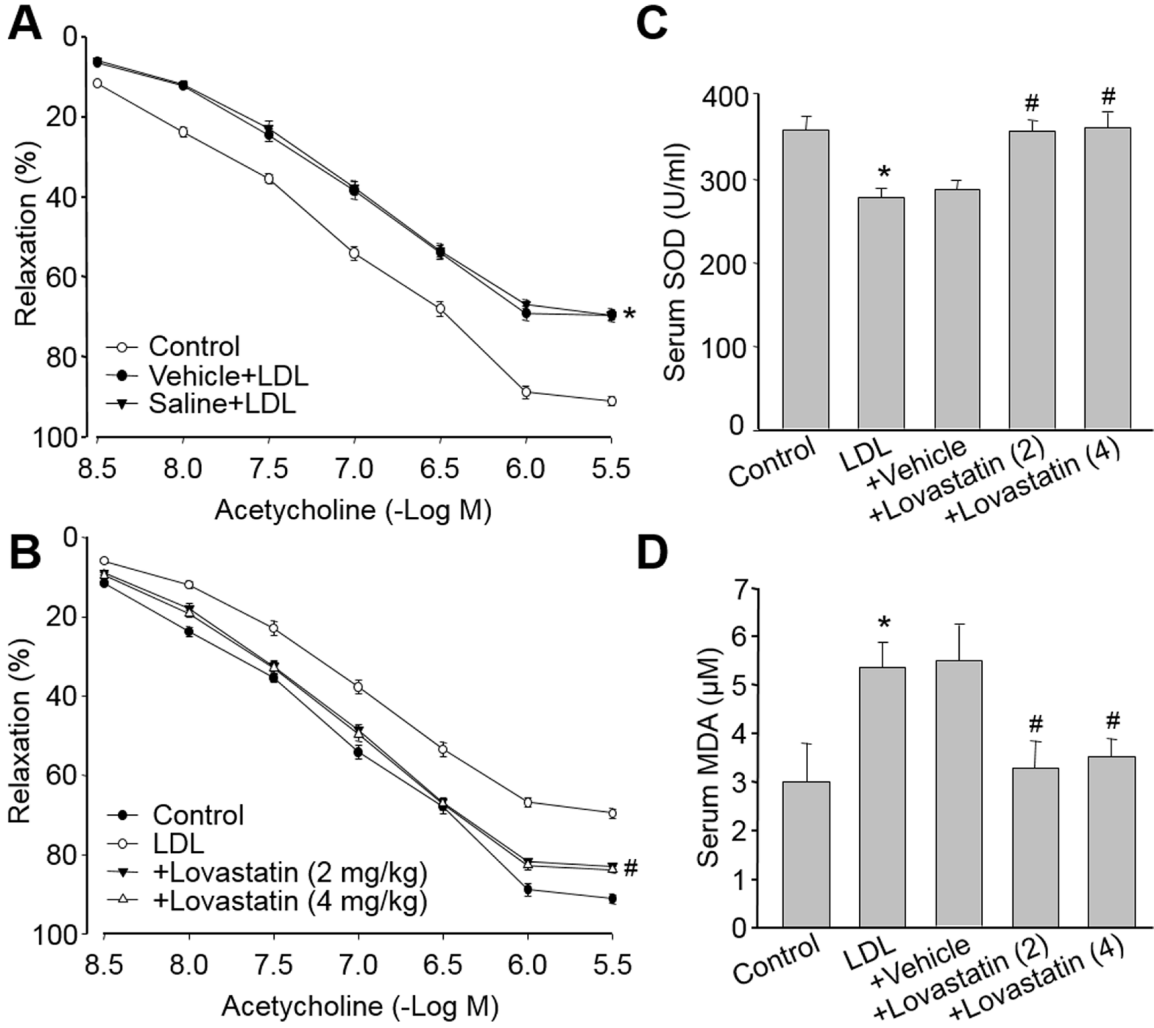
Lovastatin attenuates LDL-induced endothelial dysfunction in rats SD Rats were received treated by intraperitoneal injection with lovastatin (2 or 4 mg/kg) or vehicle once daily for 6 consecutive days followed by a single injection of n-LDL (4 mg/kg) or Saline by sublingual vein. (**A**) The endothelium- dependent relaxations induced by acetylcholine in control and LDL rats. (**B**) Does-course of lovastatin in acetylcholine-induced vasorelaxation in LDL-injected rats. (**C**) Serum SOD activity and (**D**) Serum MDA content were assayed in all rats. All data were expressed as mean ± SEM. N is 10-15 in each group. *P < 0.05 *VS* Control group. ^#^*P* < 0.05 *VS* LDL-injected rats.

We next evaluate if lovastatin prevents endothelial dysfunction *in vivo*. LDL in dyslipidemia patients which accumulates in the arterial wall is crucial to trigger the initiation and progression of atherosclerotic plaque. By using model of dyslipidemia which was established by us and others [18, 19], we found that a single injection of native LDL, but not vehicle of lovastatin or saline, markedly decreased endothelium-dependent relaxation (Figure 5A). Administration of lovastatin at 2 or 4 mg/kg/day, prior to LDL injection, rescued LDL-induced impairment of endothelial function in rats (Figure 5B). Besides, LDL-induced oxidative stress, as decreased SOD activity (Figure 5C) and increased MDA level in serum (Figure 5D), were also reversed by lovastatin treatment. Taking these data together, it demonstrates that lovastatin prevents endothelial dysfunction in dyslipidemia model, which is related to suppression of oxidative stress.

### Lovastatin improves endothelial dysfunction induced by high glucose both *ex vivo* and *in vivo*

**Figure 6:**
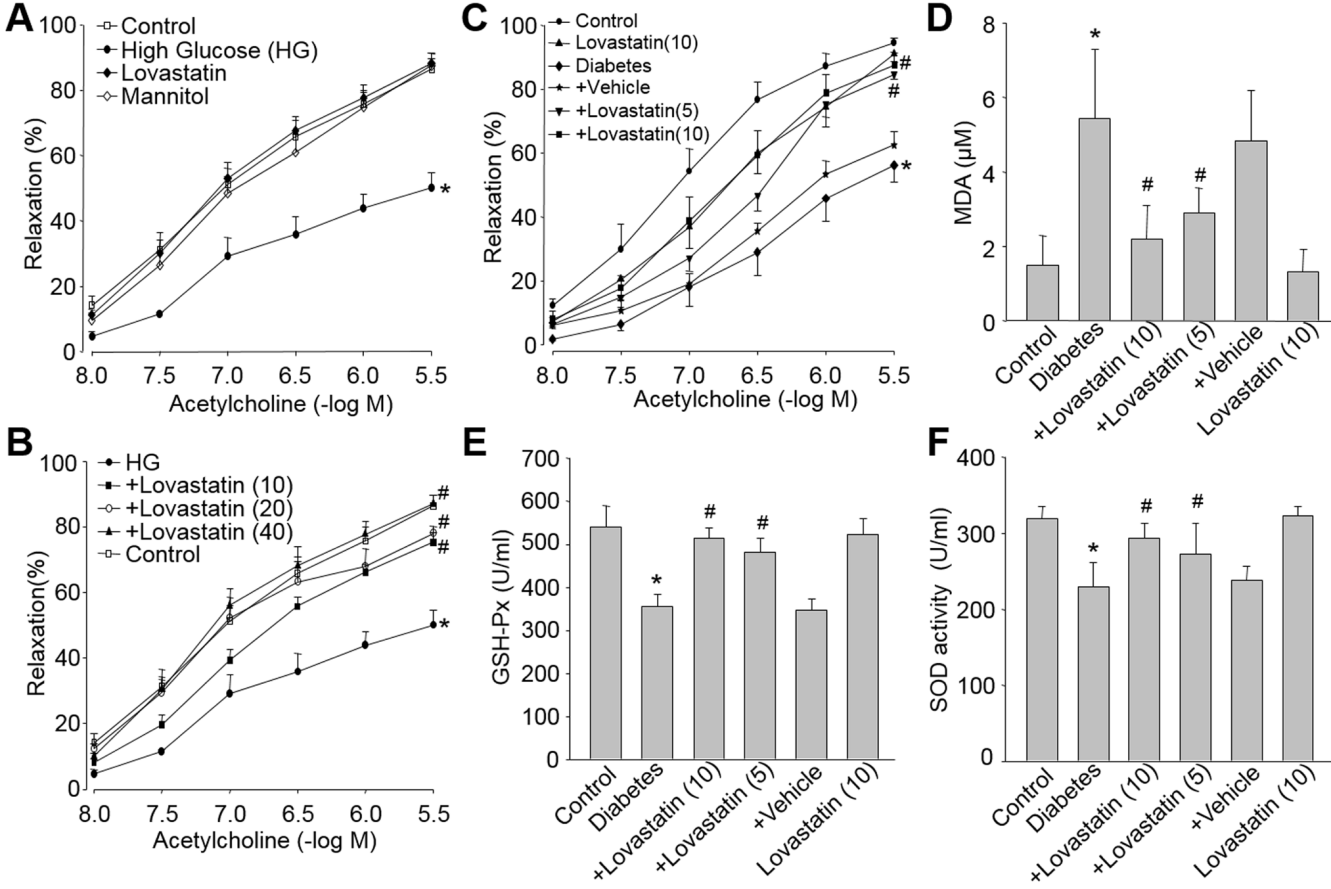
Lovastatin reduces oxidative stress and improves endothelial dysfunction induced by high glucose both *ex vivo* and *in vivo* *Ex vivo:* (**A** and **B**) Isolated aortic rings from rats were incubated with high glucose (HG, 30 mM), lovastatin (10 μM), mannitol (30 mM), HG plus lovastatin (10-40 μM). The endothelium-dependent relaxations were induced by acetylcholine in all aortic rings. All data were expressed as mean ± SEM. N is 5-10 rings in each group. *P < 0.05 *VS* Control group. ^#^*P* < 0.05 *VS* HG-treated rings. *In vivo:* (**C**-**F**) Rat models of diabetes were induced by injection of STZ (50 mg/kg/day, I.P., 5 consecutive days) and then received administration of lovastatin (5, 10 mg/kg/day) for 6 weeks. (**C**) Acetylcholine-induced vasorelaxation, (**D**) serum GSH-PX activity, (**E**) serum MDA level, and (**F**) serum SOD activity were determined. All data were expressed as mean ± SEM. N is 10-15 in each group. *P < 0.05 *VS* Control group. ^#^*P* < 0.05 *VS* Diabetes group.

We then investigated whether these actions of lovastatin on suppression of oxidative stress and protection of endothelial function would be shared by other animal models with high risk factors of CVD. To this end, we used HG or hyperglycemia model to determine the effect of lovastatin on endothelial function in diabetic rats. As indicated in Figure 6A, HG but not high osmatic decreased acetylcholine-induced vessel relaxation in isolated rat aortic rings. This was attenuated by lovastatin in dose-dependent manner (Figure 6B).

The *ex vivo* effects of lovastatin were further confirmed by *in vivo* experiments (Figure 6C). Type 1 diabetes model in rat was induced by injection of STZ. Lovastatin dose-dependently rescued the impairment of endothelial function induced by hyperglycemia. Furthermore, increased MDA level (Figure 6D) and decreased SOD and GSH-Px activities (Figure 6E and Figure 6F) in blood from diabetic rats were reversed by lovastatin treatments.

### Lovastatin normalizes hyperhomocysteinemia-induced endothelial dysfunction both *ex vivo* and *in vivo*

**Figure 7:**
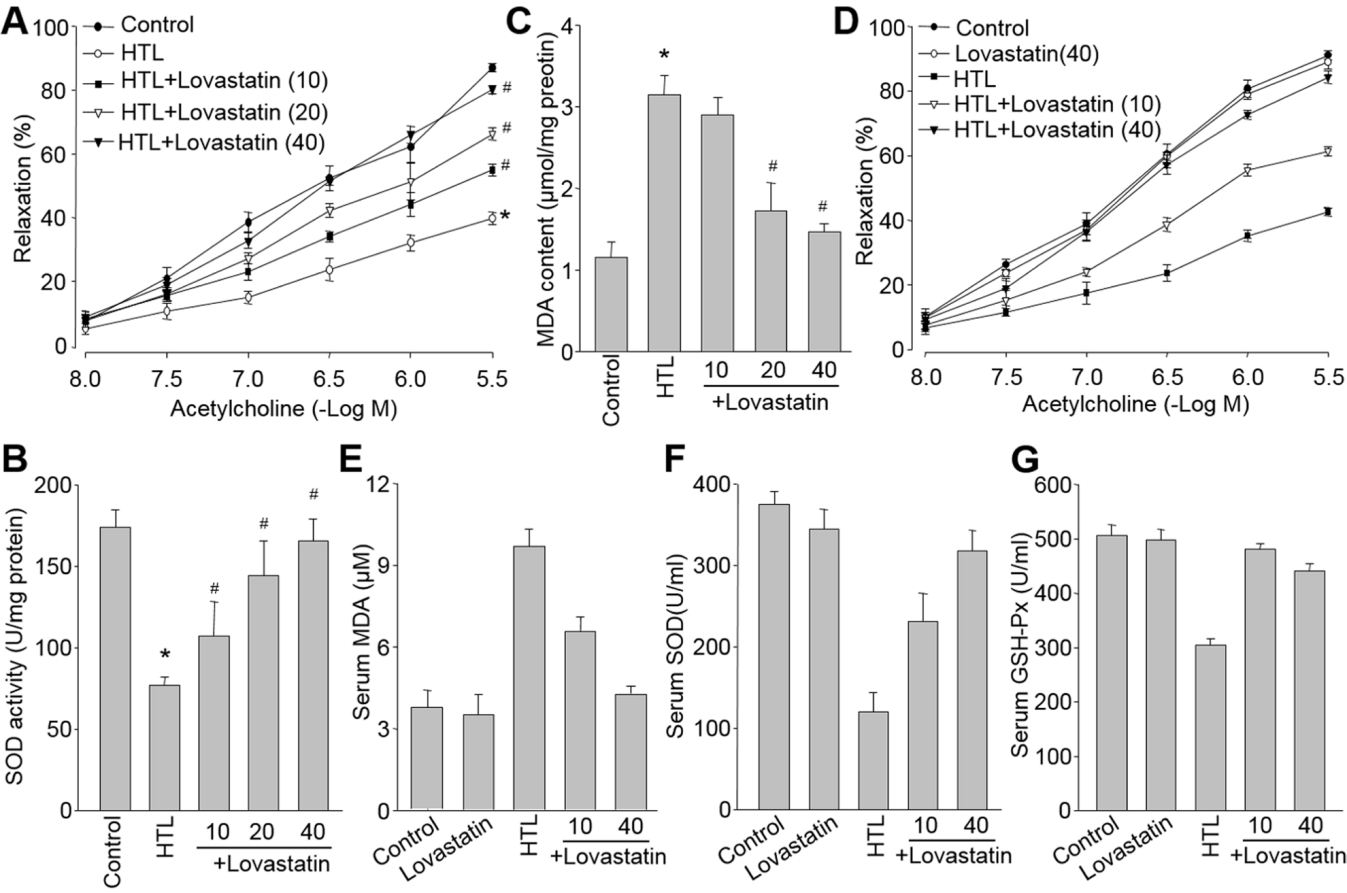
Lovastatin inhibits hyperhomocysteinemia-induced oxidative stress and endothelial dysfunction both *ex vivo* and *in vivo* *Ex vivo:* (**A**-**C**) Isolated aortic rings from rats were incubated with homocysteine thiolactone (HTL, 1 mM) with or without lovastatin (10-40 μM). (**A**) Acetylcholine-induced endothelium-dependent relaxation, (**B**) MDA content and (**C**) SOD activity in homogenates of aortic tissues were assayed. All data were expressed as mean ± SEM. N is 5-10 rings in each group. *P < 0.05 *VS* Control group. ^#^*P* < 0.05 *VS* HTL-treated rings. *In vivo:* (**D**-**G**) SD rats were intragastrically gavaged with HTL (50 mg/kg/day) and received administration of lovastatin (10, 40 mg/kg/day) for 8 weeks. (**D**) Acetylcholine-induced vasorelaxation, (**E**) serum MDA level, (F) serum SOD activity, and (**G**) serum GSH-PX activity were determined. All data were expressed as mean ± SEM. N is 10-15 in each group. *P < 0.05 *VS* Control group. ^#^*P* < 0.05 *VS* HTL group.

We also examined the effects of lovastatin on hyperhomocysteinemia model, which is an independent risk factor of CVD. We used HTL, which is the most reactive form in all of homocysteinemia metabolites [20] and has been demonstrated to induce endothelial dysfunction by us [21], to treated aortic rings isolated from SD rats. Consistently, HTL damaged endothelial function as indication of decreased endothelium-dependent relaxation (Figure 7A). Lovastatin dose-dependently prevented HTL-impaired acetylcholine-induced vasorelaxation in isolated rat aortic artery, as well as normalizations of redox status (Figure 7B and Figure 7C).

Like the effects of lovastatin in diabetes, lovastatin also eliminated all HTL-induced harmful effects in dose-dependent manner, including reduction of acetylcholine-induced vasorelaxation (Figure 7D), enhancement of serum MDA level (Figure 7E), and decreased activities of SOD and GSH-Px (Figure 7F and Figure 7G) in blood from rats fed with HTL, further supporting that prevention of endothelial dysfunction by lovastatin is a common mechanism to prevent or treat CVD induced by multiple cardiovascular risk factors.

### Lovastatin reduces ROS productions in models of diabetes, dyslipidemia, and hyperhomocysteinemia in rats

**Figure 8:**
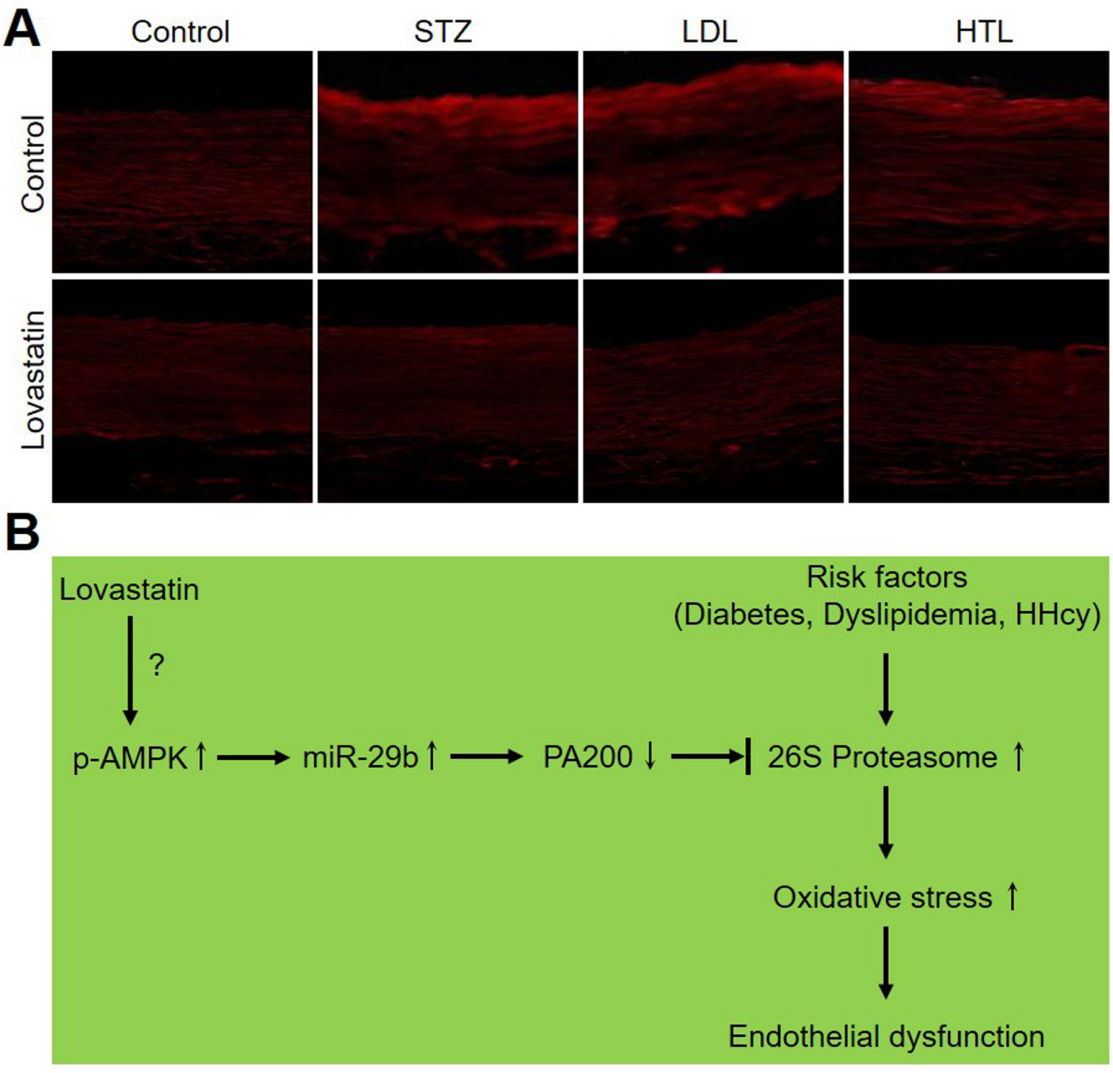
Lovastatin reduces ROS productions in models of diabetes, dyslipidemia, and hyperhomocysteinemia in rats (**A**) ROS productions were determined by staining DHE in alive carotid arteries from models of diabetes, dyslipidemia, and hyperhomocysteinemia in rats. The picture is a representative picture from 5 rats. (**B**) Proposed mechanism of lovastatin on protections of endothelial function.

Finally, we evaluated the effects of lovastatin on ROS productions in rat models with multiple cardiovascular risk factors. As shown in Figure 8A, all risk factors of CVD including diabetes, dyslipidemia, and hyperhomocysteinemia, dramatically increased ROS productions in carotid arteries from rats. Noticeably, administration of lovastatin abolished these alterations in those rats, indicating that reduction of oxidative stress by lovastatin is a common mechanism in the prevention of endothelial dysfunction in CVDs.

## DISCUSSION

In this study, we have defined a common mechanism of lovastatin to prevent endothelial dysfunction in different models of CVD, in which lovastatin-increased miR-29b expression is linked to the suppression of oxidative stress, a key step to cause endothelial dysfunction in CVD. To the best of our knowledge, this is the first report on the impacts of statin on endothelial dysfunction in multiple animal models with risk factors of cardiovascular disease.

The major finding in this paper is that lovastatin increases miR-29b expression in vascular endothelial cells, which mediates the beneficial effects of lovastatin in CVD. Statins have been reported to produce the lowering-lipid effects via inhibition of HMG-CoA reductase. In the present study, we firstly reported that lovastatin increases miR-29b expression to prevent endothelial dysfunction. Although statins have been identified to inhibit proteasome activity in many kinds of mammalian cells [12, 14, 22], including endothelial cells [11], we exploited a novel mechanism in which lovastatin increases miR-29b expression to prevent endothelial dysfunction. Emerging data support that micro-RNA, including miR-22 [23], miR-34a [24], miR-155 [25], miR-221/222 [26], is important for the multiple functions of statins, which is independent of HMG-CoA reductase inhibition. Of note, how lovastatin increases miR-29b expression in endothelial cells needs further investigations.

The study also indicates that lovastatin via suppression of oxidative stress prevents endothelial dysfunction in multiple models of CVD with high risk factors. Recently, Xu *et al* reported that oxidative stress linking to proteasome activation is a cardinal feature of endothelial dysfunction shared by different models of CVD [27]. In this study, we not only provided evidences to further support this viewpoint, but also uncover the common mechanism of lovastatin to protect against endothelial dysfunction of an early marker for multiple CVD, such as atherosclerosis and hypertension. Although statin is effective to lower blood lipid level in patients with hyperlipidemia, the functions of statin in CVD are largely controversy. In this study, we provide solid evidences, at various setting ranging from *in vitro* and *ex vivo* to whole animal models, to verify that lovastatin improves endothelial dysfunction in rats with hyperglycemia, dyslipidemia and hyperhomocysteinemia, in which all are common high risk factors of CVD. From this angle of endothelial function, statin is absolutely considerable to prevent CVD, such as atherosclerosis and hypertension.

In summary, this study reveals a novel and common mechanism of lovastatin to prevent endothelial dysfunction. As present in Figure 8B, lovastatin increases miR-29b expression to downregulate PA200 protein, resulting in proteasome inactivation and subsequent suppression of oxidative stress. In this way, lovastatin prevents endothelial dysfunction in rats with common high risk factors of CVD. Therefore, the current study will open new avenue to investigate the effects of miR-29b in CVD and also provide some insights to drug design for CVD in that targeting miR-29b to improve the outcomes of medical intervention, such as statin.

## MATERIALS AND METHODS

### Animals

Male Sprague-Dawley (SD) rats (8 ± 2 weeks old, 180 ± 20 g) were purchased from the Center of Experiment Animals, Central South University (Changsha, China). Rats were housed in temperature-controlled cages with a 12-hour light-dark cycle. This study was carried out in strict accordance with the recommendations in the Guide for the Care and Use of Laboratory Animals of the National Institutes of Health. The animal protocol was reviewed and approved by the University of Central South Animal Care and Use Committee.

### Materials

Lovastatin, lysophosphatidylcholine (LPC), hydrogen peroxide (H_2_O_2_), tumor necrosis factor alpha (TNFα), homocysteine thiolactone (HTL), D-glucose, mannitol, 1.1-dipheyl-2-picryl hydrazyl (DPPH), streptozotocin (STZ), acetylcholine, and phenylephrine were purchased from Sigma-Aldrich Company or Caymen chemical Company. Primary antibodies against PA200, p-AMPK, AMPK, and GAPDH, and secondary antibodies were obtained from Cell Signaling Company. Commercial kits malondialdehyde (MDA), superoxide dismutase (SOD) and Glutathione peroxidase (GSH-Px) were purchased from Jian-Cheng Bioengineering Institute (Nanjing, China). Oxidized low density lipoprotein (ox-LDL) was obtained from Guangzhou Yiyuan Biotech Co. Ltd. (Guangzhou, China). All drug concentrations are expressed as final working concentrations in the buffer.

### Preparation of LDL

As described previously [28], LDL was isolated by discontinuous density gradient ultracentifugation method from fresh human plasma in healthy normal-lipidemic donors. Edetic acid of 10 μM was added to the plasma to avoid autoxidation and its density was adjusted to 1.065 g/L by adding solid NaBr and the samples were ultracentrifugated (200 000×*g*, 20 h, 4°C). The purity of LDL was identified by agarose gel electrophoresis and the protein content was determined by the BCA method. The purified LDL was dialyzed for 24 h against 2 L phosphate buffer solution (pH 7.4, 0.2 M) at 4°C. Dialyzed LDL was frozen at -70°C. Before the injection of n-LDL into the animals, the LDL was sterilized after passing through a 0.45-μm filter and was kept on ice until injected.

### Cell culture

Human umbilicus vein endothelial cells (HUVECs) were obtained from Clonetics Inc. (Walkersville, MD, USA). Cells were grown in endothelial basal medium supplemented with 2% fetal bovine serum and penicillin (100 u/ml), and streptomycin (100 μg/ml). Cultured cells were used between passages 3 and 8. All cells were incubated in a humidified atmosphere of 5%CO_2_ +95% air at 37°C. When 70-80% confluent, the cells were treated with different agents.

### Generation of lentivirus vector and infection to cells

The lentivirus containing scr-miR, anti-miR29b, pre-miR29b, or full length PA200 cDNA, was generated by GenePharma Company (Shanghai, China). HUVECs were infected with lentivirus overnight in antibiotics-free medium supplemented with 2% FBS. The cells were then washed and incubated in fresh medium for an additional 12 h before experimentation as described previously [29].

### Transfection of siRNA into cells

Transient transfection of siRNA was carried out according to Santa Cruz's protocol. Briefly, the siRNAs were dissolved in siRNA buffer (20 mM KCl; 6 mM HEPES, pH 7.5; 0.2 mM MgCl_2_) to prepare a 10 μM stock solution. Cells grown in 6-well plates were transfected with siRNA in transfection medium containing liposomal transfection reagent (Lipofectamine RNAiMax, Invitrogen, Shanghai branch, China). For each transfection, 100 μl transfection medium containing 4 μl siRNA stock solution was gently mixed with 100 μl transfection medium containing 4 μl transfection reagent. After 30-min incubation at room temperature, siRNA-lipid complexes were added to the cells in 1.0 ml transfection medium, and cells were incubated with this mixture for 6 h at 37°C. The transfection medium was then replaced with normal medium, and cells were cultured for 48h.

### Establishments of diabetes, dyslipidemia, and hyperhomocysteinemia

For diabetic model of rats, a low-dose STZ (50 mg/kg/day, 5 consecutive days, I.P.) in induction regimen was used to induce pancreatic islet cell destruction and persistent hyperglycemia as previously described by the Animal Models of Diabetic Complications Consortium (http://www.amdcc.org). Hyperglycemia was defined as a random blood glucose level of > 300 mg/dl for >2 weeks after injection. The dyslipidemia model in rats has been established by us previously [18]. In brief, native LDL dissolved in saline was injected to rat (4 mg/kg) via the sublingual vein under ether anesthesia. For establishment of hyperhomocysteinemia, rats were intragastrically gavaged with HTL (50mg/kg/day) for 8 weeks with normal diet as described previously [21].

### RNA quantification

As described previously [30], total RNA was isolated using a Trizol-based (Invitrogen) RNA isolation protocol. RNA was quantified by Nanodrop (Agilent Technologies), and RNA and miRNA quality were verified using an Agilent 2100 Bioanalyzer (Agilent Technologies). First strand cDNA synthesis was performed for each RNA sample using the Superscript II RT Kit (Applied Biosystems, New York, USA). Real time quantitative polymerase chain reaction was performed using Taqman PCR reagents (Applied Biosystems), and the primers for miR-29b were designed according to the manufacturer's recommendations. The relative amount of miRNA was standardized against U6 snRNA, and the fold change for miR-29b was calculated using the 2-DD computed tomography method.

### Proteasome activity assay

Cells were washed with PBS and then with buffer I (50 mM Tris, pH 7.4, 2 mM DTT, 5 mM MgCl_2_, 2 mM ATP). The cells were then made into pellets by centrifugation. Homogenization buffer (50 mM Tris, pH 7.4, 1 mM DTT, 5 mM MgCl_2_, 2 mM ATP, 250 mM sucrose) was added, and cells were subjected to a vortex for 1 minute. Cell debris was removed by centrifugation at 1000g for 5 minutes followed by 10 000g for 20 minutes. Protein (100 μg) from each sample was diluted with buffer I to a final volume of 1000 μl. The fluorogenic proteasome substrate Suc-LLVY-7-amido-4-methylcoumarin was added at a final concentration of 80 μM in 1% DMSO. Cleavage activity was monitored continuously by detection of free 7-amido-4-methylcoumarin with a fluorescence plate reader (Gemini, Molecular Devices, Sunnyvale, CA, USA) at 380/460 nm at 37°C.

### Western blotting

As described previously [31], aortic tissues were homogenized on ice in cell-lysis buffer (20 mM Tris-HCl, pH 7.5, 150 mM NaCl, 1 mM Na_2_EDTA, 1 mM EGTA, 1% Triton, 2.5 mM sodium pyrophosphate, 1 mM beta-glycerophosphate, 1 mM Na_3_VO_4_, 1 μg/ml leupeptin) and 1 mM PMSF. Cell was lysated with cell-lysis buffer. The protein content was assayed by BCA protein assay reagent (Pierce, USA). 20 μg proteins were loaded to SDS-PAGE and then transferred to membrane. Membrane was incubated with a 1:1000 dilution of primary antibody, followed by a 1:2000 dilution of horseradish peroxidase- conjugated secondary antibody. Protein bands were visualized by ECL (GE Healthcare). The intensity (area × density) of the individual bands on Western blots was measured by densitometry (model GS-700, Imaging Densitometer; Bio-Rad). The background was subtracted from the calculated area. We used control as 100%.

### Detection of ROS productions in cells and tissues

ROS productions in cultured cells were assayed by measuring the DHE fluorescence, combined with High Performance Liquid Chromatography (HPLC) with minor modifications as describe previously [32]. Briefly, cells were incubated with DHE (10 μM) for 30 min, washed, harvested and homogenized, and subjected to methanol extraction. HPLC was performed by using a C-18 column (mobile phase: gradient of acetonitrile and 0.1% trifluoroacetic acid) to separate and quantify oxyethidium (product of DHE and O_2_^-^) and ethidium (a product of DHE auto-oxidation). O_2_^-^ production was determined by conversion of DHE into oxyethidine.

To measure ROS production in the artery *in situ*, fresh frozen sections of carotid arteries were isolated from rats, and were stained with 10 μM DHE for 30 min, rinsed, and observed by fluorescent microscopy. Results were quantified using BIOQUANT Image software.

### Measurement of tension development in aortic rings

*In vivo* or *ex vivo* organ chamber study was performed as described previously [33]. Rats were sacrificed under anesthesia by intravenous injection with pentobarbital sodium (30 mg/kg). The descending aorta isolated by removing the adhering perivascular tissue carefully was cut into rings (3-4 mm in length). Aortic rings were suspended and mounted to organ chamber by using two stainless. The rings were placed in organ baths filled with Kreb's buffer of the following compositions (in mM): NaCl, 118.3; KCl, 4.7; MgSO_4_, 0.6; KH_2_PO_4_, 1.2; CaCl_2_, 2.5; NaHCO_3_, 25.0; EDTA, 0.026; pH 7.4 at 37°C and gassed with 95% O_2_ plus 5% CO_2_, under a tension of 2.0 g, for 90-minute equilibration period. During this period, the Kreb's solution was changed every 15 min. After the equilibration, aortic rings were challenged with 60 mM KCl. After washing and another 30 minutes equilibration period, contractile response was elicited by phenylephrine (1 μM). At the plateau of contraction, accumulative acetylcholine was added into the organ bath to induce the relaxation.

### Measurements of MDA, SOD and GSH-Px

The determinations of MDA content, SOD activity and GSH-Px activity in aortic tissues or serum were assayed by using commercial kits as recommend by the protocol.

### Statistical analysis

All quantitative results are expressed as mean ± s.e.m. The vessel responses to Ach or SNP are expressed as percentages of pre-contractions and these data were analyzed using a two-way ANOVA followed by Bonferroni corrections. One-way ANOVA was used to compare multiple groups followed by Newman-Keuls student t-tests. Statistical analysis was conducted using IBM SPSS statistics 20.0 (IBM Corp., Armonk, NY, USA) and *P* < 0.05 were considered as statistical significance.

## SUPPLEMENTARY DATA SET



## References

[1] Kozarov E, Padro T, Badimon L View of statins as antimicrobials in cardiovascular risk modification. Cardiovasc Res.

[2] Wang S, Peng Q, Zhang J, Liu L Na+/h+ exchanger is required for hyperglycaemia-induced endothelial dysfunction via calcium-dependent calpain. Cardiovasc Res.

[3] Yang J J, Li P, Wang F, Liang W J, Ma H, Chen Y, Ma Z M, Li Q Z, Peng Q S, Zhang Y, Wang S X Activation of activator protein 2 alpha by aspirin alleviates atherosclerotic plaque growth and instability in vivo. Oncotarget.

[4] Wang S, Zhang M, Liang B, Xu J, Xie Z, Liu C, Viollet B, Yan D, Zou M H Ampkalpha2 deletion causes aberrant expression and activation of nad(p)h oxidase and consequent endothelial dysfunction in vivo: Role of 26s proteasomes. Circ Res.

[5] Wang S, Xu J, Song P, Viollet B, In Zou M H vivo activation of amp-activated protein kinase attenuates diabetes-enhanced degradation of gtp cyclohydrolase i. Diabetes.

[6] Liu H, Yu S, Xu W, Xu J Enhancement of 26s proteasome functionality connects oxidative stress and vascular endothelial inflammatory response in diabetes mellitus. Arterioscler Thromb Vasc Biol.

[7] Khor B, Bredemeyer A L, Huang C Y, Turnbull I R, Evans R, Maggi L B, White J M, Walker L M, Carnes K, Hess R A, Sleckman B P Proteasome activator pa200 is required for normal spermatogenesis. Mol Cell Biol.

[8] Ustrell V, Hoffman L, Pratt G, Rechsteiner M Pa200, a nuclear proteasome activator involved in DNA repair. EMBO J.

[9] Tsimokha A S, Kulichkova V A, Karpova E V, Zaykova J J, Aksenov N D, Vasilishina A A, Kropotov A V, Antonov A, Barlev N A DNA damage modulates interactions between micrornas and the 26s proteasome. Oncotarget.

[10] Jagannathan S, Vad N, Vallabhapurapu S, Vallabhapurapu S, Anderson K C, Driscoll J J Mir-29b replacement inhibits proteasomes and disrupts aggresome+autophagosome formation to enhance the antimyeloma benefit of bortezomib. Leukemia.

[11] Ludwig A, Friedel B, Metzkow S, Meiners S, Stangl V, Baumann G, Stangl K Effect of statins on the proteasomal activity in mammalian endothelial and vascular smooth muscle cells. Biochem Pharmacol.

[12] Murray S S, Tu K N, Young K L, Murray E J The effects of lovastatin on proteasome activities in highly purified rabbit 20 s proteasome preparations and mouse mc3t3-e1 osteoblastic cells. Metabolism.

[13] Hisada T, Ayaori M, Ohrui N, Nakashima H, Nakaya K, Uto-Kondo H, Yakushiji E, Takiguchi S, Terao Y, Miyamoto Y, Adachi T, Nakamura H, Ohsuzu F, Ikewaki K, Sakurai Y Statin inhibits hypoxia-induced endothelin-1 via accelerated degradation of hif-1alpha in vascular smooth muscle cells. Cardiovasc Res.

[14] Rao S, Porter D C, Chen X, Herliczek T, Lowe M, Keyomarsi K Lovastatin-mediated g1 arrest is through inhibition of the proteasome, independent of hydroxymethyl glutaryl-coa reductase. Proc Natl Acad Sci U S A.

[15] Park I H, Kim J Y, Choi J Y, Han J Y Simvastatin enhances irinotecan-induced apoptosis in human non-small cell lung cancer cells by inhibition of proteasome activity. Invest New Drugs.

[16] Choi H C, Song P, Xie Z, Wu Y, Xu J, Zhang M, Dong Y, Wang S, Lau K, Zou M H Reactive nitrogen species is required for the activation of the amp-activated protein kinase by statin in vivo. J Biol Chem.

[17] Blickwedehl J, Agarwal M, Seong C, Pandita R K, Melendy T, Sung P, Pandita T K, Bangia N Role for proteasome activator pa200 and postglutamyl proteasome activity in genomic stability. Proc Natl Acad Sci U S A.

[18] Ma F X, Liu L Y, Xiong X M Protective effects of lovastatin on vascular endothelium injured by low density lipoprotein. Acta Pharmacol Sin.

[19] Calara F, Dimayuga P, Niemann A, Thyberg J, Diczfalusy U, Witztum J L, Palinski W, Shah P K, Cercek B, Nilsson J, Regnstrom J An animal model to study local oxidation of ldl and its biological effects in the arterial wall. Arterioscler Thromb Vasc Biol.

[20] Malinowska J, Kolodziejczyk J, Olas B The disturbance of hemostasis induced by hyperhomocysteinemia; the role of antioxidants. Acta Biochim Pol.

[21] Yang X H, Li P, Yin Y L, Tu J H, Dai W, Liu L Y, Wang S X (2015). Rosiglitazone via ppargamma-dependent suppression of oxidative stress attenuates endothelial dysfunction in rats fed homocysteine thiolactone. J Cell Mol Med.

[22] Wojcik C, Bury M, Stoklosa T, Giermasz A, Feleszko W, Mlynarczuk I, Pleban E, Basak G, Omura S, Jakobisiak M Lovastatin and simvastatin are modulators of the proteasome. Int J Biochem Cell Biol.

[23] Tu Y, Wan L, Bu L, Zhao D, Dong D, Huang T, Cheng Z, Shen B Microrna-22 downregulation by atorvastatin in a mouse model of cardiac hypertrophy: A new mechanism for antihypertrophic intervention. Cell Physiol Biochem.

[24] Tabuchi T, Satoh M, Itoh T, Nakamura M Microrna-34a regulates the longevity-associated protein sirt1 in coronary artery disease: Effect of statins on sirt1 and microrna-34a expression. Clin Sci (Lond).

[25] Sun H X, Zeng D Y, Li R T, Pang R P, Yang H, Hu Y L, Zhang Q, Jiang Y, Huang L Y, Tang Y B, Yan G J, Zhou J G Essential role of microrna-155 in regulating endothelium-dependent vasorelaxation by targeting endothelial nitric oxide synthase. Hypertension.

[26] Minami Y, Satoh M, Maesawa C, Takahashi Y, Tabuchi T, Itoh T, Nakamura M Effect of atorvastatin on microrna 221 / 222 expression in endothelial progenitor cells obtained from patients with coronary artery disease. Eur J Clin Invest.

[27] Xu J, Wang S, Zhang M, Wang Q, Asfa S, Zou M H Tyrosine nitration of pa700 links proteasome activation to endothelial dysfunction in mouse models with cardiovascular risk factors. PLoS One.

[28] Gonzalez-Timon B, Gonzalez-Munoz M, Zaragoza C, Lamas S, Melian E M Native and oxidized low density lipoproteins oppositely modulate the effects of insulin-like growth factor i on vsmc. Cardiovasc Res.

[29] Wang S, Liang B, Viollet B, Zou M H Inhibition of the amp-activated protein kinase-alpha2 accentuates agonist-induced vascular smooth muscle contraction and high blood pressure in mice. Hypertension.

[30] Maegdefessel L, Azuma J, Toh R, Merk D R, Deng A, Chin J T, Raaz U, Schoelmerich A M, Raiesdana A, Leeper N J, McConnell M V, Dalman R L, Spin J M, Tsao P S Inhibition of microrna-29b reduces murine abdominal aortic aneurysm development. J Clin Invest.

[31] Wang S, Zhang C, Zhang M, Liang B, Zhu H, Lee J, Viollet B, Xia L, Zhang Y, Zou M H Activation of amp-activated protein kinase alpha2 by nicotine instigates formation of abdominal aortic aneurysms in mice in vivo. Nat Med.

[32] Wang S, Xu J, Song P, Wu Y, Zhang J, Chul Choi H, Zou M H Acute inhibition of guanosine triphosphate cyclohydrolase 1 uncouples endothelial nitric oxide synthase and elevates blood pressure. Hypertension.

[33] Wang J, Guo T, Peng Q S, Yue S W, Wang S X Berberine via suppression of transient receptor potential vanilloid 4 channel improves vascular stiffness in mice. J Cell Mol Med.

